# Autosomal recessive variants c.953A>C and c.97-1G>C in NSUN2 causing intellectual disability: a molecular dynamics simulation study of loss-of-function mechanisms

**DOI:** 10.3389/fneur.2023.1168307

**Published:** 2023-05-25

**Authors:** Nazif Muhammad, Syeda Iqra Hussain, Zia Ur Rehman, Sher Alam Khan, Samin Jan, Niamatullah Khan, Muhammad Muzammal, Sumra Wajid Abbasi, Naseebullah Kakar, Zia Ur Rehman, Muzammil Ahmad Khan, Muhammad Usman Mirza, Noor Muhammad, Saadullah Khan, Naveed Wasif

**Affiliations:** ^1^Department of Biotechnology and Genetic Engineering, Kohat University of Science and Technology (KUST), Kohat, Khyber Pakhtunkhwa, Pakistan; ^2^Department of General Medicine, Northwest General Hospital & Research Center, Peshawar, Khyber Pakhtunkhwa, Pakistan; ^3^Gomal Center of Biochemistry and Biotechnology, Gomal University, D.I.Khan, Khyber Pakhtunkhwa, Pakistan; ^4^NUMS Department of Biological Sciences, National University of Medical Sciences, The Mall, Rawalpindi, Punjab, Pakistan; ^5^Department of Biotechnology, Faculty of Life Sciences and Informatics, BUITEMS, Quetta, Pakistan; ^6^Institute of Human Genetics, Universitätsklinikum Schleswig-Holstein, Lübeck, Germany; ^7^Department of Chemistry and Biochemistry, University of Windsor, Windsor, ON, Canada; ^8^Institute of Human Genetics, Ulm University, and Ulm University Medical Center, Ulm, Germany; ^9^Institute of Human Genetics, University Hospital Schleswig-Holstein, Kiel, Germany

**Keywords:** consanguinity, *NSUN2* gene, intellectual disability, novel variants, molecular dynamics simulation

## Abstract

**Introduction:**

Intellectual disability (ID) is a clinically and genetically heterogeneous disorder. It drastically affects the learning capabilities of patients and eventually reduces their IQ level below 70.

**Methods:**

The current genetic study ascertained two consanguineous Pakistani families suffering from autosomal recessive intellectual developmental disorder-5 (MRT5). We have used exome sequencing followed by Sanger sequencing to identify the disease-causing variants.

**Results and discussion:**

Genetic analysis using whole exome sequencing in these families identified two novel mutations in the *NSUN2* (NM_017755.5). Family-A segregated a novel missense variant c.953A>C; p.Tyr318Ser in exon-9 of the *NSUN2*. The variant substituted an amino acid Tyr318, highly conserved among different animal species and located in the functional domain of *NSUN2* known as “SAM-dependent methyltransferase RsmB/NOP2-type”. Whereas in family B, we identified a novel splice site variant c.97-1G>C that affects the splice acceptor site of *NSUN2*. The identified splice variant (c.97-1G>C) was predicted to result in the skipping of exon-2, which would lead to a frameshift followed by a premature stop codon (p. His86Profs^*^16). Furthermore, it could result in the termination of translation and synthesis of dysfunctional protein, most likely leading to nonsense-mediated decay. The dynamic consequences of *NSUN2* missense variant was further explored together with wildtype through molecular dynamic simulations, which uncovered the disruption of *NSUN2* function due to a gain in structural flexibility. The present molecular genetic study further extends the mutational spectrum of *NSUN2* to be involved in ID and its genetic heterogeneity in the Pakistani population.

## 1. Introduction

Intellectual disability (ID) is a severe manifestation of the central nervous system leading to drastically reduced general mental abilities, intelligent working, and adaptive behavior compared with people of the same age group, gender, and sociocultural setup. The primary distinguishing characteristics of intellectual disability involve IQ (intelligence quotient) < 70, impairment in at least two adaptive abilities, and disease onset before 18 years of age (1, 2). The epidemiological estimate indicates that 1–3% of the general population is affected by intellectual disability. In addition to consanguinity, developing countries are also facing the issues of malnutrition, lack of health facilities, unhygienic environment, and cultural deprivation as contributing risk factors of intellectual disability. Furthermore, the recent literature survey has reported over 700 genetic entities involved in syndromic and non-syndromic ID (3) and are transmitted in an autosomal dominant, recessive, X-Linked, or mitochondrial fashion (Orphanet report). The autosomal recessive inheritance of intellectual disability is relatively rare and accounts for < 12% of cases of intellectual disabilities (4). Physiologically, the reported ID genes are involved in various cellular signaling cascades, inter-neuronal connectivity, proliferation, and migration, and the genetic or epigenetic control over transcription and translation of the genes (5).

The *NSUN2* (NOP2/Sun RNA methyltransferase 2) gene is located at 5p15, comprising 19 exons. The gene transcribes a 2.1-kb mRNA molecule expressed in brain tissue and generates a methyltransferase enzyme. The tRNA precursors' cytosine at positions 34 and 48 is methylated to 5-methylcytosine (m^5^C) (6). This modification might promote tRNA stability and mRNA export (7–9), and enzymatic failure is associated with many diseases, such as autism spectrum disorder (10), depression (11), Dubowitz syndrome (12, 13), and non-syndromic autosomal recessive intellectual disability (ARID) (14, 15), and is differentially expressed in various cancers (16–19). Evidence for the significance of NSUN2-mediated m^5^C in ID has been recently reported in Pakistani, Iranian, Qatari, and Lebanese families (9, 13, 20–22). NSUN2 is shown to be localized to the nucleoli of Purkinje cells, and it has been found that these nucleoli are often situated between or in close proximity to dense heterochromatic regions (9).

Over the past decade, advances in NGS techniques and machine learning development have sped up gene identification and increased the diagnostic efficiency of rare genetic disorders, revolutionizing the healthcare systems (23). This genetic study aimed at identifying disease-causing variants in two unrelated Pashtun families of Pakistani origin containing five affected members with impairments in adaptive behavior and cognitive functioning. We have used exome sequencing and Sanger sequencing to identify the genetic variations.

With the advancement in molecular modeling campaigns, molecular dynamics simulations are considered an adequate method to explore the dynamic effects on protein structure due to mutations (16, 24–27), which consented to investigate the conformational characteristics (28, 29). Our findings after molecular dynamics (MD) simulations delineate the structural insights of novel disease-causing variant and provide a molecular illustration of plausible aberrations caused by mutation.

## 2. Materials and methods

### 2.1. Sample collection

**Figure 1:**
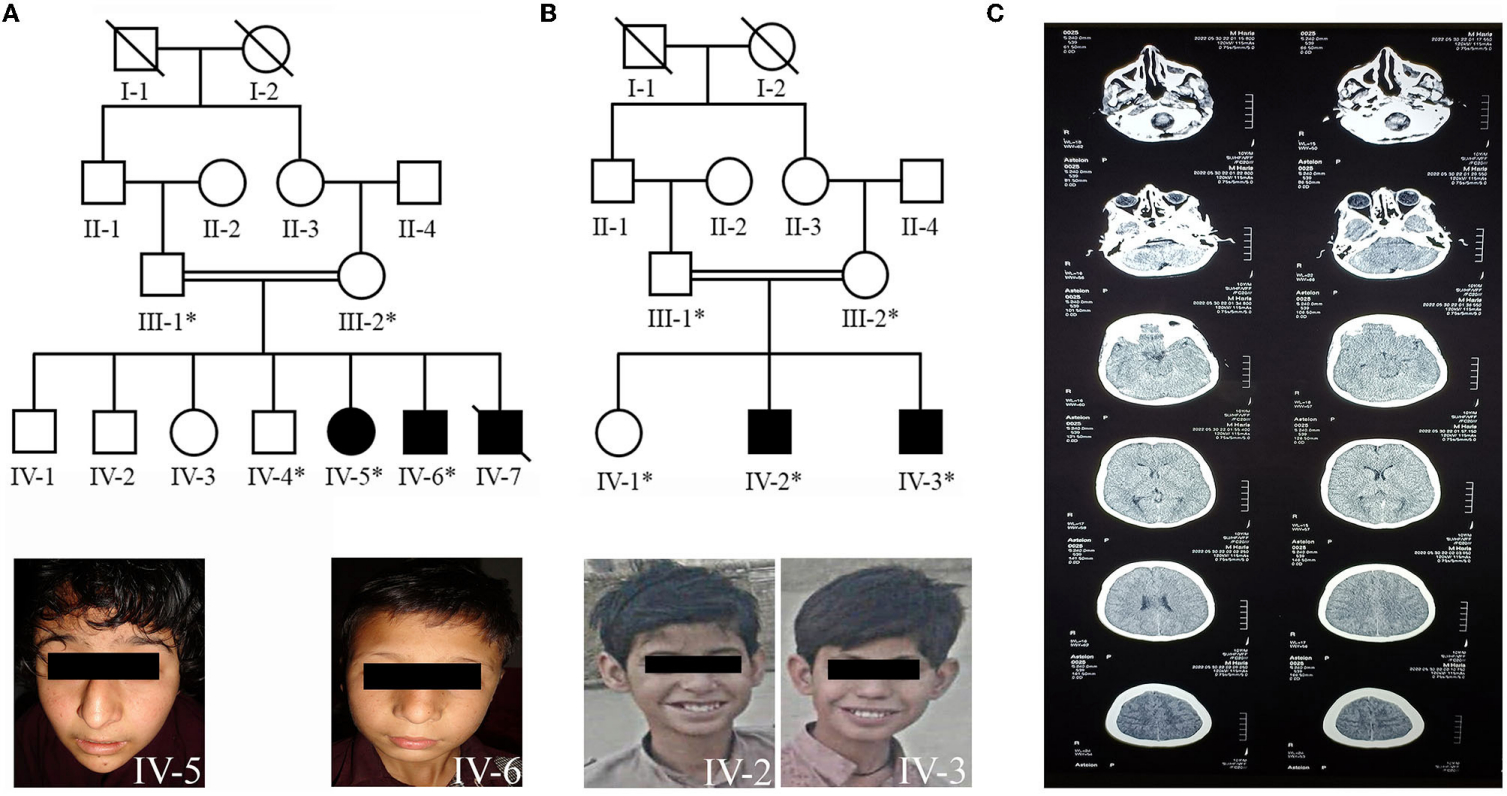
**(A)** Pedigree of family A showing autosomal recessive inheritance of non-syndromic ID and the patients (IV-5, IV-6) facial photographs. **(B)** Pedigree of family B demonstrating the autosomal recessive inheritance of non-syndromic ID and the patients (IV-2, IV-3) facial photographs. **(C)** Showing the computed tomography (CT) scan image of the patient (IV-3) of family B.

In this study, two consanguineous families (family A and family B), segregating autosomal recessive intellectual disability, were ascertained from Upper Dir and district Karak of Khyber Pakhtunkhwa (KP) province in Pakistan. Familial-based sampling approach recruited two individuals affected with ID, and three unaffected members, including parents from each family (Figures 1A, B). The institutional ethical review board of Kohat University of Science and Technology, Kohat, approved this study, and patients were enrolled after written informed consent. Blood samples were collected in EDTA Vacutainer tubes, and DNA was extracted using the standard phenol–chloroform method.

### 2.2. Exome sequencing and variant calling in family A

Exome sequencing was performed for a paired-end library using the Nextera DNA Exome kit (Illumina Inc., USA) as per the manufacturer's instructions. Approximately 100 ng genomic DNA of an index patient (IV-5) was used for the enzymatic fragmentation, subsequently performing unique adaptors ligation (dual indexes) and exome enrichment. Exome sequencing (75 × 2 paired-end) was performed using NextSeq^®^ 550/500 High Output Reagent Cartridge v2 kit on Illumina NextSeq500 instrument (Illumina, San Diego, CA, USA).

The filtration of primary data was carried out by the Illumina real-time analysis (RTA) software v1.8. Afterward, the BWA-SW alignment algorithm (30) was used to map the reads to the human reference genome build GRCh37/hg19 (http://www.genome.ucsc.edu/). Using Samtools v.0.0.1.9 (31), the subsequent sequence alignment map file (SAM) was converted into a binary alignment map (BAM) format. Picard tools (32) were used to improve the read quality, and Genome Analysis Toolkit (GATK) was used for realignment and base quality score recalibration (33). The calling of single nucleotide polymorphisms (SNPs) and short insertions/deletions (INDELs) was performed by Platypus (34), Haplotype Caller (35), and Mpileup programs (31), and further filtration was carried out through variant quality score calibration (VQSR) using GATK (33). For the coverage analysis of CNV detection, CNMOPS and ExomeDepth algorithms were utilized (36, 37). In addition, the COMBINE and FUNC algorithms (varpipe_v2.26, https://varbank.ccg.uni-koeln.de/) combined the data and the annotation of functional variants.

### 2.3. Data analysis and variant filtration

Exome sequencing data analysis and filtration of variants were performed using the exome analysis pipeline “Varvis” (Limbus Medical Technologies GmbH). The mean coverage of the data at 20X and 10X of the targeted bases was >92 and 96.6%, respectively. In addition, we performed exclusion mapping of homozygous or compound heterozygous variants in OMIM genes associated with autosomal recessive intellectual disability. We analyzed the exome sequencing data for variants with coverage of >6 reads and an allele frequency ≥75% in the affected individual. Furthermore, a minor allele frequency (MAF) < 0.5% in the gnomAD database was observed. The filtration criteria identified 10 homozygous variants [*RPL5*: c.891C>A, p.(Ser297Arg), *DYNC2I1*: c.845C>T, p.(Ser282Leu), *HEMK1*: c.157G>C, p.(Glu53Gln), *ITIH1*: c.2515G>A, p.(Gly839Ser), *ITIH1*: c.2510_2511insTAGAAGTTTCTGA, p.(Ile838Argfs^*^4), *PFKP*: c.1490C>T, p.(Thr497Met), *USP19*: c.1447G>T, p.(Gly483Cys), *VAV1*: c.2411A>C, p.(Lys804Thr), *MBTPS1*: c.493G>C, p.(Ala165Pro)], including a homozygous variant (c.953A>C, p.Tyr318Ser) in *NSUN2*. An in-house intellectual disability gene panel, which includes autosomal dominant, recessive, and X-linked genes involved in syndromic and non-syndromic intellectual disability, did not reveal any other rare variant except the *NSUN2* variant.

### 2.4. Exome sequencing and variant calling in family B

Genomic DNA was extracted from the patient's blood samples. First, the exonic regions of all 22,000 human genes were captured by the xGen Exome Research Panel v2 (Integrated DNA Technologies, Coralville, Iowa, USA). After capture, NovaSeq 600 was used to sequence all captured regions (Illumina, San Diego, CA, USA). We acquired ≥20X coverage in >98.9% and ≥10X coverage in >99.1% of target sequences.

Following the sequencing, the data were analyzed using open-source bioinformatics tools and proprietary software. bcl2fastq v2.20.0.422 (https://emea.support.illumina.com/downloads/bcl2fastq-conversion-software-v2-20.html) was used to convert and demultiplex base call (BCL) sequence files to FASTQ files. Variant calling and annotation followed the alignment of the sequencing data to the GRCh37/hg19 human reference genome was carried out using BWA-mem 0.7.17 (arXiv:1303.3997 [q-bio.GN]) to generate BAM files. BAM files were processed using GATK best practices (GATK v.3.8, broadinstitute.org) for single nucleotide variants (SNV) and small insertions/deletions (indel) variant calling to generate VCF files (33, 38). For copy number variant (CNV) calling based on depth-of-coverage (DOC) data, Conifer (39) and 3bCNV (https://3billion.io/resources) are used. The region of homozygosity (ROH) was mapped from the VCF file using AutoMap v1.2 (40).

One of the in-house tools, EVIDENCE, was designed to select variants based on ACMG guidelines and each patient's phenotype. Variant filtration, categorization, and similarity score for the patient's phenotype are three significant steps in this approach. For allele frequency estimation, a genome aggregation database (gnomAD, http://gnomad.broadinstitute.org/) and a 3-billion genome database were utilized in the first step. According to ACMG guidelines, gene variations with more than 5% allele frequency were filtered out. Next, the VarSome (41), Human Gene Mutation Database (HGMD) Professional 2022.1, Database of Single Nucleotide Polymorphisms (dbSNP), and ClinVar (https://www.ncbi.nlm.nih.gov/clinvar/) were utilized for the evaluation of variants. Then, the assessment of each variant concerning disease phenotype was carried out using the ACMG guidelines (42). Finally, in the third step, the patient's clinical phenotypes were converted to standardized human phenotype ontology terms (https://hpo.jax.org/) and retrieved to determine the degree of similarity (43, 44) with each of 7,000 rare genetic diseases (https://omim.org/ and https://www.orpha.net/consor/cgi-bin). According to the ACMG guideline, the similarity score between each patient's phenotype and symptoms related to that disease caused by priority variations varied from 0 to 10. The filtration criteria revealed four rare variants (*RELN*: c.8843+3A>C, *PMM2*: c.348-5C>A, *MADCAM1*: c.784_785insAGGAGCCTCCCGACACCACCTCCCAGGAGCCTCCCGACACCACCTCCC, p.Ser261_Pro262insGlnGluProProAspThrThrSerGlnGluProProAspThrThrSer, *NSUN2*: c.97-1G>C).

### 2.5. Segregation analysis and sequence conservation alignment

The filtered variants were then subjected to Sanger sequencing to confirm variant segregation with disease phenotype in the whole family with the available DNA samples. Primers (flanking the variant regions) were designed using the online Primer3 software. We used the following primer sets for the missense variant (c.953A>C) “forward primer: 5'-GGACTGGAATGTATGATACCAA-3' and reverse primer: 5'-AATAGAACGGTGGTGTGAGG-3”' and, for splice acceptor site variant (c.97-1G>C) “forward primer: 5'-AGGTGTAGGGCTAGAGTTCTG-3' and reverse primer: 5'-CTCAATGCTTCCTGAATCC-3”'.

Multiple protein sequence alignment was performed through the Clustal Omega tool (Embl-Ebi Clustal Omega) to check the conservation of identified missense variant https://www.ebi.ac.uk/Tools/msa/clustalo/ (accessed August 30, 2022). The pathogenicity of the novel missense variant of *NSUN2* was predicted through SIFT (45), PolyPhen 2.0 (46), and I-Mutant 3.0 (47).

### 2.6. Molecular modeling and protein stability predictions

Although no x-ray-resolved crystal structure of *NSUN2* is available in the protein data bank, the homology modeling was performed using human-*NSUN6* complexed with tRNA and S-adenosylmethionine (SAM). Both share a strikingly similar architecture; therefore, the user-defined template method of SWISS-MODEL was executed to build the wild-type (*wt*) and mutant (*mt*) *NSUN2* model, which extracts initial structural information from the template structure (48). As the inherent structural elucidation relies on homology model, the reliability of the *NSUN2* model was evaluated using MolProbity (49) and ProSA (50) and further refined and optimized through MD simulations. In addition, protein function and the stability effect upon substitution (ΔΔG) were assessed using the DUET server (51, 52), a combined computational approach that combines two complementary techniques (mCSM and SDM). Moreover, the thermal stability (ΔΔG) rising from vibrational entropy changes (ΔΔS) was also predicted using the Elastic Network Contact Model (ENCoM) server (53). Finally, the effect of mutation on the overall structural dynamics of *wt*-*NSUN2* compared with its wild-type (*wt*) was analyzed through MD simulations, which were performed in two steps: 100 ns MD simulation to refine and optimize the models (*wt* and *mt*), while another 100 ns to analyze the residual fluctuations of *NSUN2* with or without reported mutation. All simulations were executed using AMBER 20 (54) using the same protocol we described elsewhere (25, 55). Furthermore, the figures were drawn using Chimera (56), while structure-based protein multiple sequence alignment of *NSUN* members (1–3, 5–8) was generated by ESPript (57).

## 3. Results

### 3.1. Clinical features of family A

Family A resides in the Upper Dir area of Khyber Pakhtunkhwa. The family was Pashtun by ethnicity, who usually preferred cousin marriages. The family's pedigree consists of four generations, in which the affected individuals (IV-5, IV-6, and IV-7, Figure 1A) born to first-cousin parents appeared in the fourth generation. Before our first sampling visit, patient IV-7 was deceased due to unknown reasons. There was no prior family history of any genetic disease.

Clinical features of all the affected individuals from family A had moderate-to-severe ID with speech cluttering and unable to understand the arithmetic calculations. The patients had relatively small stature, abnormal behavioral features, and developmental delay. However, no epileptic fits, spasticity, hypotonia, or deep tendon reflexes were observed. Both patients exhibited dysmorphic facial features with ptosis, shallow and flat philtrum grooves, thin upper and thick lower lips, high nasal bridges, and protruding teeth in common. A pointed chin, heart-shaped face, and snub nose with droopy nasal tip were peculiar to the female patient (IV-5). The male patient (IV-6) also had additional dysmorphic facial features, including a square-shaped face, hypertelorism, sparse eyebrows, and a big nose (Figure 1A, IV-5, IV-6).

### 3.2. Clinical features of family B

Family B also belonged to Pashtun ethnic group and resided in District Karak. However, it is unrelated to family A. The presented family pedigree consisted of four generations with two affected individuals (IV-2 and IV-3, Figure 1B) also born to first consanguineous parents.

Clinical features of the affected individuals from family B include moderate-to-severe ID with speech cluttering and facial dysmorphism. The prominent facial dysmorphic features included square-shaped faces, thick scalp hair, ptosis, large ears, shallow philtrum grooves, thin upper and thick lower lips, and prominent teeth (Figure 1B, IV-2, IV-3). They had no counting or recognition abilities. They could not recognize and differentiate among different currency notes. They also had a lack of social interactions and often showed aggressive modes upon irritation. The patient's (IV-3) computed tomography (CT) scan image found evidence of fluid present bilaterally in the maxillary and ethmoid sinuses of the brain (Figure 1C). The additional clinical description of patients in both families is summarized in Supplementary Table 1.

### 3.3. Molecular findings

**Figure 2:**
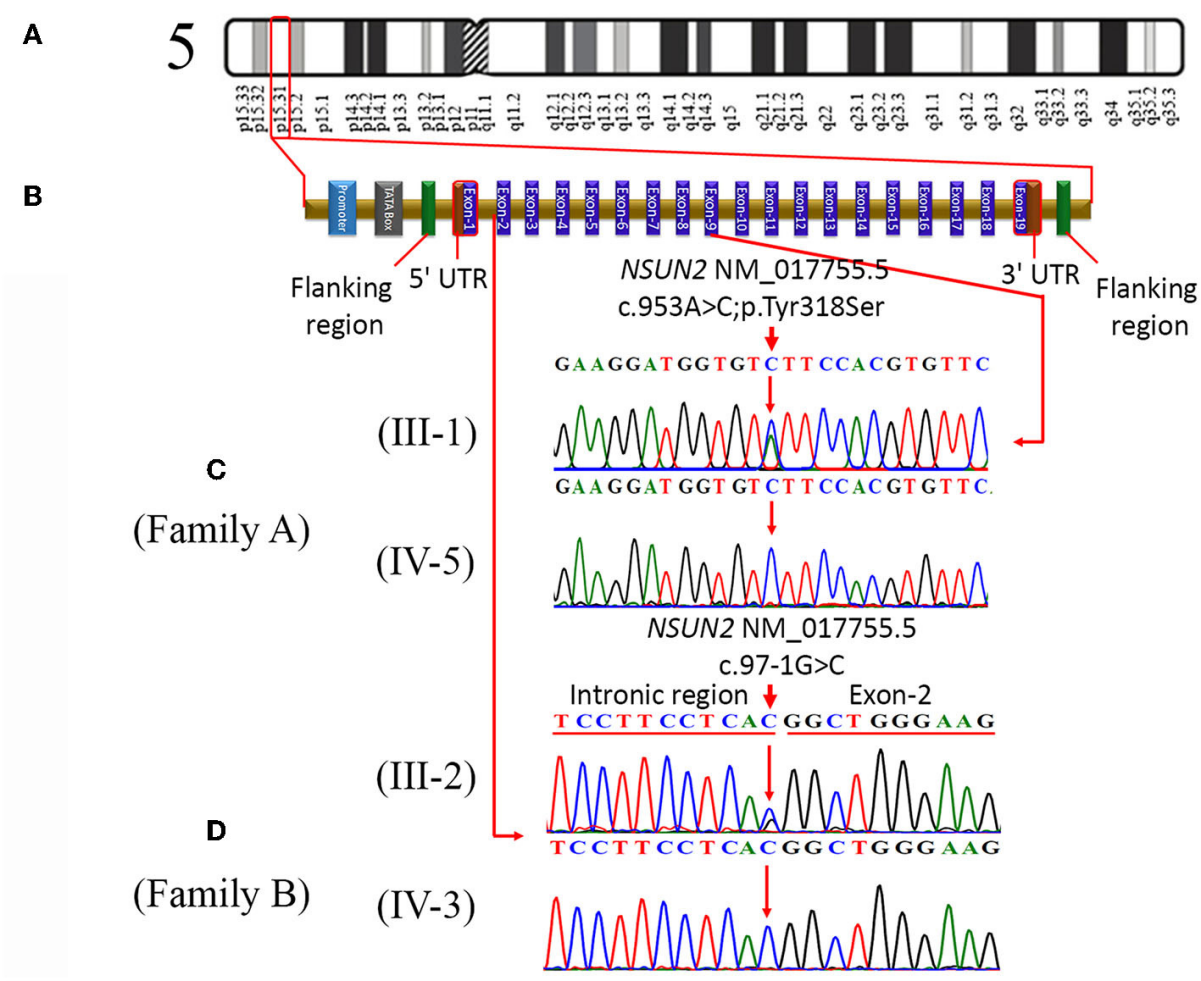
**(A)** Represents cytogenic location (p15.31) of *NSUN2* on chromosome 5, **(B)** typical structure of *NSUN2* gene, consisting of 19 exons. It also indicates the location of a missense variant (c.953A>C; p.Tyr318Ser) in exon-9 and the location of a splice acceptor site variant (c.97-1G>C) in the intronic region. **(C)** Sequence chromatograms of a heterozygous father (III-1) and homozygous affected daughter (IV-5) in family A. **(D)** Partial sequence chromatograms of the heterozygous mother (III-2) and homozygous affected son (IV-3) in family B.

Genetic analysis using whole exome sequencing performed on the DNA of an affected individual from family A identified 10 homozygous variants, including a missense variant (c.953A>C, p.Tyr318Ser) in *NSUN2* (Figures 2A, B). Sanger sequencing validated and confirmed the segregation of only *NSUN2* variant with the disease phenotype in the family. Furthermore, the zygosity analysis determined that the affected individuals (IV-5 and IV-6) were homozygous (c.953C/C). At the same time, the unaffected parents (III-1 and III-2), as well as the unaffected sibling (IV-4), were heterozygous carriers (c.953A/C) for the identified missense variant (Figure 2C), consistent with an autosomal recessive mode of inheritance.

In family B, exome sequencing of a patient (IV-2) revealed a novel homozygous splice site variant (c.97-1G>C) in the *NSUN2* (Figure 2B), along with the other three variants on different chromosomal locations. Likewise, in segregation analysis in family A, the segregation of the *NSUN2* variant with disease phenotype was also confirmed by Sanger sequencing. Zygosity analysis found that the unaffected sibling (IV-1) and both unaffected parents (III-1 and III-2) were heterozygous c.97-1G/C, while both the affected individuals (IV-2 and IV-3) were homozygous c.97-1C/C for splice site variant (Figure 2D). The nomenclature of both variants is based on the transcript NM_017755.5. The variants identified other than *NSUN2* variants were excluded during the segregation analysis.

### 3.4. Structural elucidation and MD interpretations

**Figure 3:**
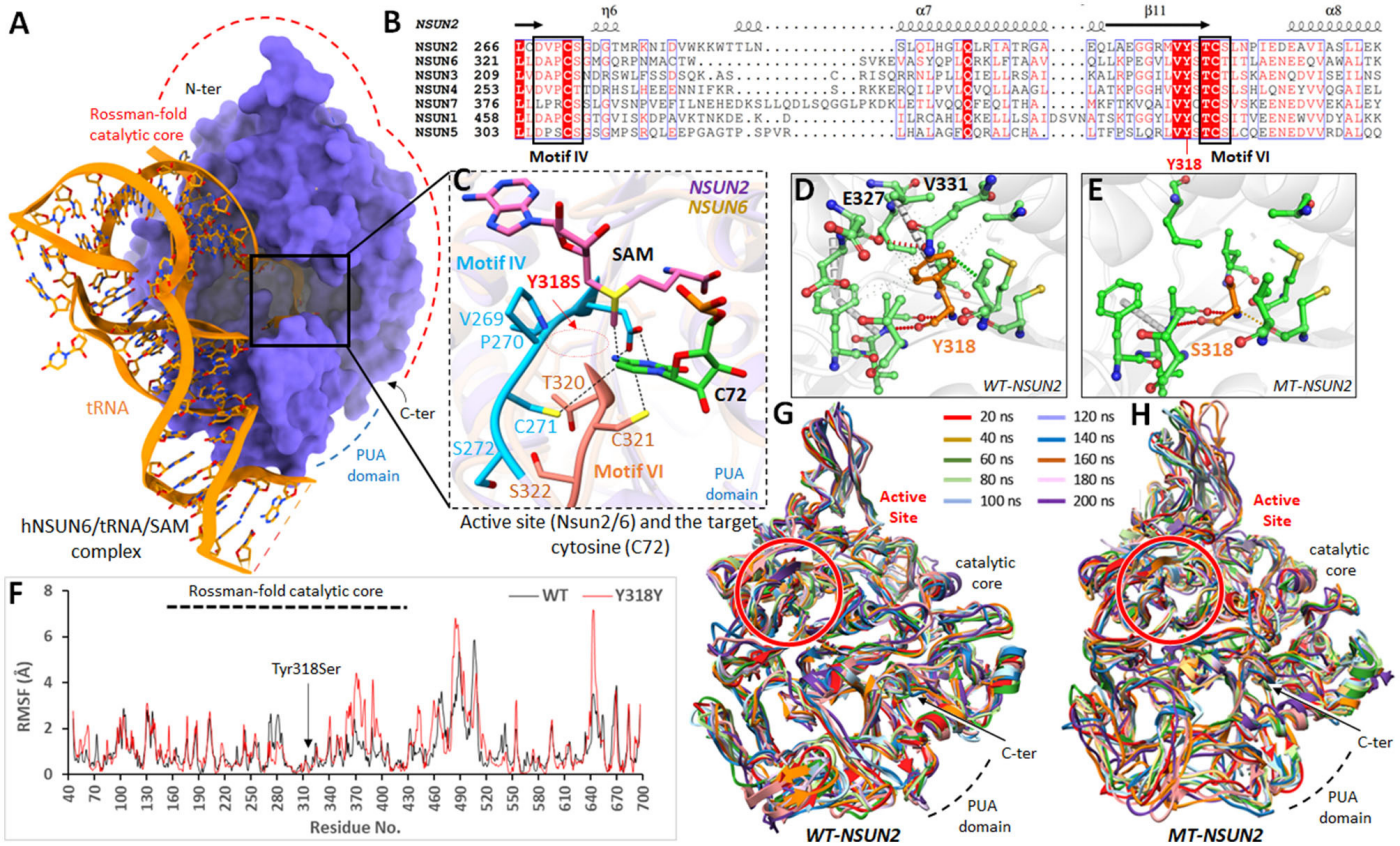
Molecular modeling analysis of NSUN2 upon Tyr318Ser mutation. **(A)** Overall structure of hNSUN6/tRNA/SAM complex (PDB 5WWS) with catalytic core and PUA domain are highlighted. **(B)** Amino acid sequence alignment of regions forming the active sites of human m^5^C methyltransferases belong to the NSUN family. Conserved motifs IV and VI are boxed, and the location of the mutation is labeled red. **(C)** The active site of NSUN2 is superimposed on NSUN6/tRNA/SAM complex, showing the arrangement and crucial contacts between the active site residues and the target cytosine (C72) in RNA. **(D)** Wild-type (Tyr318) and **(E)** mutant residues (Ser318) of NSUN2 are colored orange (as sticks) alongside the surrounding residues, which are involved in interactions. **(F)** RMSF trajectories of mutant (red) and wild-type (blue) NSUN2 are displayed, representing the per-residue fluctuation during molecular dynamics (MD) simulations. **(G, H)** Structural conformations of mutant and wild-type NSUN2 models displayed, obtained after every 20 ns over 200 ns, are superimposed together.

SWISS-MODEL generated the *NSUN2* models with high accuracy due to the template belonging to the human *NSUN6*, which revealed a sequence identity of 34.32 and query coverage of 0.61. The model is refined and optimized for 100 ns MD simulations. Later, the homology model evaluations determined 90.21% residues in Ramachandran favored regions, and ProSA determined the reliable z-score (-7.5) as the overall quality model (see Supplementary Figure S1). Like in *NSUN6*, the *NSUN2* revealed a similar Rossmann-fold catalytic core (residues 171–429) and PUA domain (residues 54–147) that accommodates the SAM cofactor when superimposed on *NSUN6/tRNA/SAM*. In addition, the *NSUN* proteins use two catalytic cysteines in the active site, present in conserved motif IV (Cys271 in *NSUN2*) and VI (Cys321 in *NSUN2*) (Figures 3A–C).

In *NSUN6/tRNA/SAM*, the methylation target C72 has profound interactions with *NSUN6* and the methylation factor, SAM. The side chains of Lys248 (190 in *NSUN2*), Asp323 (motif IV; 268 in *NSUN2*), Cys326 (motif IV; 271 in *NSUN2*), Cys373 (motif VI; 321 in *NSUN2*), and Phe458 (422 in *NSUN2*) and the main chain of residue Ser371 (319 in *NSUN2*) are all involved in recognition of C72. The conformations of these C72 and SAM interacting residues were strikingly similar in *NSUN2*. The Tyr318Ser mutation resided in the conserved antiparallel β-sheets structural fold of *NSUNs*, in the vicinity of active site (tRNA/SAM binding site) (Figure 3B; multiple sequence alignment**)**. To examine the dynamic consequences of Tyr318Ser mutation in the active site, the protein structure stability changes (ΔΔG) upon mutation in the *mt*-*NSUN2* model were estimated from various programs. DUET predicted Tyr318Ser in *NSUN2* as destabilizing with a negative free energy change (ΔΔG) value of −3.694 kcal/mol. Moreover, the estimated vibrational entropy energy change (ΔΔS_Vib_) and thermal stability (ΔΔG), as accessed from ENCoM server (53), also indicated the increase in protein flexibility (ΔΔS_Vib_ ENCoM: 1.127 kcal.mol^−1^.K^−1^; ΔΔG: −4.077 kcal/mol as destabilizing). The calculated ΔΔS per residue vibrational entropy changes indicated the region (residues 260–330), which gained flexibility upon Tyr318Ser substitution, while Ser318 revealed ΔΔS of −0.254 kcal.mol^−1^.K^−1^ (see Supplementary Figure S2).

Tyr318 was present in critical central core region close to active site and found crucial in establishing significant non-covalent interactions and buttressed the configuration of conserved *NSUN* fold inside the catalytic core. The substitution to Ser318 abolished these crucial interactions, especially the amide-ring (pi-pi) interaction between the backbone amide of Val331 and benzene ring of Tyr318 (Figures 3D, E), which instigated and amplified the flexibility toward the active site of *NSUN2* as predicted from vibrational entropy energy. To prove the hypothesis of increased flexibility in *NSUN2* upon Tyr318Ser substitution at an atomistic level, 100 ns MD simulations were executed on *wt* and *mt*-*NSUN2* models. The structural stability of the Rossmann catalytic core and PUA domain was investigated during simulations by computing mainly the root mean square fluctuations (RMSF) concerning the wild-type structures. RMSF graph highlighted the flexible regions of *wt* and *mt*-*NSUN2*.

Over a simulation period of 100 ns, residual fluctuations of the catalytic core retained within 3Å in *wt*-*NSUN2*, and the structure remained converged (Figure 3F). In contrast, mt-*NSUN2* revealed evident Cα-RMSF differences in the catalytic core for residues 338–400 and amplified the fluctuations for residues 460–510. In addition, replacing Tyr318 with Ser318 elicited substantial mobility over simulation period, which retained its impact on the adjacent regions, including the compact active site core indicated to accommodate the methylation factor and facilitate the binding with C72. Overall, the fluctuations revealed the extent of the conformational shift of various regions, including the active site in the *mt-NSUN2* compared with its *wt*, which remained stable and converged throughout the simulation (Figures 3G, H).

## 4. Discussion

Subcellular, NSUN2 is localized in the nucleolus of cells (9). Physiologically, NSUN2 is involved in the post-transcriptional modification of tRNA and is a well-defined RNA methyltransferase responsible for cytosine-5 methylation (m^5^C) of tRNA-Leu (CAA) at position C34 (58). CCA is a conserved sequence at the 3′ end of mature tRNA-Leu molecules to function as the site of leucine attachment. This sequence is added and maintained by tRNA nucleotidyltransferase (59). NSUN2 was first reported in non-syndromic autosomal recessive ID by Khan et al. (9) and Abbasi-Moheb et al. (22) in consanguineous Iranian and Pakistani families in 2012 (9, 22). The first SUN-domain-containing protein discovered in vertebrates was NSUN2, which is highly conserved from bacteria to humans. Frye and Watt identified it as a modulator of Myc-induced proliferation as it is the human homolog of the yeast Trmt4 protein. They also discovered that it exhibits methyltransferase activity against rRNA, tRNA, and hemimethylated DNA *in vitro*. The structural integrity, translational efficiency, and fidelity of tRNAs are all affected by methylation and other modifications (60). The nucleotide in the wobble position of tRNA-leu is modified by NSUN2 (CAA). The functional loss of NSUN2 could result in the absence of tRNA-leu (CAA) and lead to changes in tissue-specific protein production, such as changes in codon usage. Codon usage is known to significantly impact translation rate in mammals, particularly during cell differentiation (61). NSUN2 may have a significant role in translational control during synaptic plasticity; thus, its functional loss may affect learning and memory.

Nsun2^−/−^-knockout mice showed a significant reduction in body weight and body size in two Nsun2-knockout lines, as reported by Blanco et al. (62). These findings match the short stature observed in several affected individuals, including patients from the present study and previously reported families by Khan et al. (9). In addition, while working on a mouse model, Khan et al. said that NSUN2 protein was localized to the nucleolus of Purkinje cells in the cerebellum of the mouse. The effects of the pathogenic variant (p.Gly679Arg) were confirmed by the transfection of wild-type and mutant constructs into cells and subsequent immunohistochemistry. In addition, p.Gly679Arg caused the failure of NSUN2 localization within the nucleolus (9).

*NSUN2* genetic variations are reported in several consanguineous families, mainly from Pakistan or Iran. Primarily, Najamabadi and his colleagues in 2007 found the MRT5 locus at chromosome 5p15.31 associated with the ID in two Iranian families. Later, Abbasi-Moheb and co-workers in 2012 identified two nonsense variants (c.679C>T, p. Gln227Ter; c.1114C>T, p.Gln372Ter) in *NSUN2* located within MRT5 region 16, 20, 21. Another group of researchers working on a Kurdish family also identified a homozygous transversion (g.6622224A>C) located 11-bp upstream from exon-6. This genomic variant predictably causes premature termination (Ile179ArgfsTer192) in exon-7 due to the skipping of exon-6 (22). Similarly, a Lebanese family reported a splice site mutation adjacent to exon-6. This splice site change (G>C transversion) occurred at 1-bp upstream of exon-6 of *NSUN2* (13). Finally, in the same year, a missense variant c.2035G>A (p.Gly679Arg) in exon-19 of *NSUN2* was reported in a Pakistani family, which resulted in a mutant protein that failed to localize appropriately in the nucleolus (9).

Apart from non-syndromic ID, *NSUN2* variants are also involved in Noonan-like and Dubowitz-like syndromes. Nonetheless, *NSUN2* variants in non-syndromic patients also exhibit some commonly occurring abnormal features, such as moderate to severe ID, short stature, facial dysmorphism with a long face, distinctive eyebrows, high nasal bridge or broad nasal base, drooping eyes, small pointed chin, and speech cluttering (62). The detailed description of all reported pathogenic variations and their associated clinical features are summarized in Supplementary Table 2.

Our current genetic study enrolled two consanguineous Pakistani families with Pashtun ethnicity segregating non-syndromic ID. Genetic analysis in family A revealed a novel missense variant (c.953A>C; p.Tyr318Ser) in exon-9 of the *NSUN2*, while in family B, a novel splice site variant (NM_017755.5, c.97-1G>C) in the same gene was identified. The identified novel splice variant (p.His86Profs^*^16) introduced a premature termination codon, expected to cause a non-sense mediated decay (NMD), leading to the loss of function of NSUN2 protein. However, Hentze and Kulozik (63) reported that the imperfect messages initiated by truncating mutations are eliminated by NMD (63).

In this study, the impact of the Tyr318Ser novel missense variant on the stability of *NSUN2* was accessed through a series of computational methods to improve the predictions. The possible effects due to Tyr318Ser substitution include (i) the considerable decrease in the side chain volume, which could eliminate the electrostatic interactions with the neighboring side chains (64), (ii) shifts in the side-chain packing, and (iii) changes in solvent accessibility, which has a crucial role in secondary structure stabilization (65, 66), thus destabilizing the overall active site conformation. Furthermore, MD simulation revealed substantial fluctuations in NSUN2 in several regions upon Tyr318Ser substitution, especially in the β-sheet catalytic core region.

The fact that flexibility causes large fluctuations in *mt-NSUN2* suggests that a slight conformational change upon mutation stimulates a considerable structural change in the peripheral region of the protein (67). This inherent occurrence of flexibility might significantly destabilize the overall active site configuration in *mt-NSUN2*, which is necessary to recognize methylation target C72 and conformation of a cofactor for methylation activity. For example, Asp293 (268 in *NSUN2*) recognizes the N6 of the adenine ring, and its displacement could lead to the loss of SAM binding capability and a loss of methylation activity to tRNA^Cys^. Likewise, removing Ser371 (319 in NSUN2; one residue ahead to the mutation site), typically involved in recognizing C72, might disrupt this interaction. Additionally, these dynamic consequences were evident from increased vibrational entropy energy between *wt* and *mt-NSUN2* (ΔΔS_Vib_: 1.127 kcal.mol^−1^.K^−1^), which worsened the flexibility of catalytic core [calculated by ENCoM (53)], as observed in the contiguous residues toward both directions from the site of substitution (Supplementary Figure S2). This vibrational entropy considerably influences the binding free energies of proteins (53, 68). Therefore, we speculated that the Tyr318Ser substitution of NSUN might be a loss-of-function mutation that eventually leads to the loss of SAM binding capability and methylation activity to tRNA^Cys^. Overall, the *NSUN2* structural analyses uncovered that this pathogenic variant alters the overall protein function and implicates the abrupt catalytic mechanism of the RNA:m5C methyltransferase, thus leading to non-syndromic ID.

## 5. Conclusion

The present molecular genetic study found two novel variants in *NSUN2* and, thus, further extended the mutational spectrum and allelic heterogeneity of *NSUN2* and its association with non-syndromic ID. Furthermore, the comprehensive genotype-linked phenotype analysis has found that the *NSUN2* variant usually leads to facial dysmorphism, moderate ID, short stature, and speech delay. The long-term ID conditions of the patients create numerous social barriers. Such genetic investigations not only increase general awareness about the disease but also help to improve personalized patient support and treatment.

## Data availability statement

The original contributions presented in the study are publicly available. This data can be found here: National Center for Biotechnology Information (NCBI) ClinVar, https://www.ncbi.nlm.nih.gov/clinvar/, SCV002599111 and SCV002599110.1.

## Ethics statement

The studies involving human participants were reviewed and approved by Institutional Ethical Review Board of Kohat University of Science and Technology, Kohat, Pakistan. Written informed consent to participate in this study was provided by the participants' legal guardian/next of kin. Written informed consent was obtained from the individual(s), and minor(s)' legal guardian/next of kin, for the publication of any potentially identifiable images or data included in this article.

## Author contributions

NaM and SH have performed experiments and data analysis. SK, SJ, and NKh recruited the families. ZR (3rd author) performed clinical analysis, while MMu, SA, and ZR (10th author) were involved in Sanger sequencing. MMi performed molecular modeling studies. NKa, MK, NoM, NW, and SK conceptualized and supervised the study and remained involved in data analysis, manuscript drafting, and fund acquisition. All authors have read, edited, and approved the final version of the manuscript.
