# Supplementary material for: Autosomal recessive variants c.953A>C and c.97-1G>C in NSUN2 causing intellectual disability: a molecular dynamics simulation study of loss-of-function mechanisms
Source: Front Neurol. 2023 May 25;14:1168307. doi: 10.3389/fneur.2023.1168307 (PMC10249782; doi:10.3389/fneur.2023.1168307)
Supplement: Supplementary file 1 [file Table_1.DOCX]

Table 1: Clinical features of patients suffering from *NSUN2* mutation.

| **Clinical features** | **Family A** | | **Family B** | |
| --- | --- | --- | --- | --- |
| Causative gene | NSUN2 | | NSUN2 | |
| cDNA | 953A<C | 953A<C | c.97-1G>C | c.97-1G>C |
| Protein variant | Tyr318Ser | Tyr318Ser | - | - |
| Patient ID | IV:3 | IV:6 | IV:2 | IV:3 |
| Gender | Female | Male | Male | Male |
| General Physique | Relatively short structure | Relatively short structure | Relatively short structure | Relatively short structure |
| Disease onset | Congenital | Congenital | Congenital | Congenital |
| Speech disability | Yes | Yes | Yes | Yes |
| Developmental Delay | Present | Present | Present | Present |
| Intellectual disability | Present | Present | Present | Present |
| Seizure or Epileptic fitz | Not Observed | Not Observed | Not Observed | Not Observed |
| Arithmetic skills | No | No | No | No |
| Spasticity | Not Observed | Not Observed | Not Observed | Not Observed |
| Hypotonia | Not Observed | Not Observed | Not Observed | Not Observed |
| Deep tendon reflexes | Not Observed | Not Observed | Not Observed | Not Observed |
| Behavioural abnormalities | Present | Present | Present | Present |
| Long Face | Yes | Yes | Yes | Yes |
| High Nasal Bridge | Yes | Yes | No | No |
| Ptosis | Yes | Yes | Yes | Yes |
| Protruding teeth | Yes | Yes | Yes | Yes |
| Renal Abnormality | No | No | No | No |
| Micro- or Macrocephaly | Not found | Not found | Not found | Not found |
| Poly- or Syndactyly | No | No | No | No |
| Obesity | No | No | No | No |
| Gait Problems | No | No | No | No |
| Movement of joint | Normal | Normal | Normal | Normal |
