# Supplementary material for: Autosomal recessive variants c.953A>C and c.97-1G>C in NSUN2 causing intellectual disability: a molecular dynamics simulation study of loss-of-function mechanisms
Source: Front Neurol. 2023 May 25;14:1168307. doi: 10.3389/fneur.2023.1168307 (PMC10249782; doi:10.3389/fneur.2023.1168307)
Supplement: Supplementary file 2 [file Table_2.DOCX]

**Supplementary Table 2:** Allelic heterogeneity of *NSUN2* and its associated clinical consequences

| **S. No** | **Mutation** | **ID** | **Microcephaly** | **Short Stature** | **Facial dysmorphism** | **Broad/depressed nasal bridge** | **Gait** | **Muscle deformities** | **Speech Delay** | **Reference** |  |
| --- | --- | --- | --- | --- | --- | --- | --- | --- | --- | --- | --- |
| **Missense mutation** | | | | | | | | | | | |
| 1 | c.2035G>A;p.Gly679Arg | Moderate | Yes | Yes | Yes | Yes | Broad | Yes | Yes | ^6^ |  |
| 2 | c.593 T > G;p. Lys198Arg | Severe | Yes | Yes | Yes | Yes | - | Yes | Yes | ^1^ |  |
| **Nonsense mutation** | | | | | | | | | | | |
| 3 | c.679C>T;p.Gln227Ter | Moderate | Yes, in one patient and not in two patients | Yes | Yes | Yes | - | Yes | - | ^19, 55^ |  |
| 4 | c.1114C>T;p.Gln372Ter | Moderate | Yes | Yes | Yes | Yes | - | Yes | - | ^19^ |  |
| 5 | c.1004T>A;p.Leu335Term | Moderate | Yes | Yes | Yes | Yes | - | Yes | Yes | ^56^ |  |
| **Frameshift mutation** | | | | | | | | | | | |
| 6 | g.6622224A>C;p.Ile179ArgfsTer192 | Moderate | Yes, in one patient and not in two patients | Yes | Yes | Yes | - | Yes | - | ^19, 57^ |  |
| 7 | c.1020delA;p.Gly341Valfs*15 | Moderate | Yes | Yes | Yes | Yes | Broad | Yes | - | ^58^ |  |
| 8 | c.1373delA;p.Asn458Ilefs*18, g.6607343delT | - | Yes | Yes | Yes | - | - | - | - | ^59^ |  |
| 9 | c.546_547insCT,;p.Met183Leufs*13 | Moderate to severe | Yes | Yes | Yes | Yes | Yes | Yes | Yes | ^60^ |  |
| 10 | c.1583del;p.Pro528Hisfs*19 | Moderate to severe | Yes | Yes | Yes | Yes | Yes | Yes | Yes | ^60^ |  |
| 11 | c.1269- dup;p.Val424Cysfs*14 | Moderate to severe | Yes | Yes | Yes | Yes | Yes | Yes | Yes | ^60^ |  |
| 12 | c.1478delA;p.Asn496Ilefs*18 | Moderate | Yes | Yes | Yes | Yes |  | Yes | - | ^59^ |  |
| **Splice site** | | | | | | | | | | | |
| 13 | c.1095+1G>A | Moderate | Yes | Yes | Yes | Yes | - | Yes | - | ^61^ |  |
| 14 | c.433-124T>G | Moderate | Yes | Yes | Yes | Yes | - | Yes | - | ^19^ |  |
| 15 | c.360-1G>C (IVS3- 1G>C), c.787G>T;p.Asp263Tyr | Moderate to severe | Yes | Yes | Yes | Yes | Yes | Yes | - | ^62^ |  |
| 16 | 1-bp deletion (c.915del) | Noonan-like syndrome | | | | | | | - | ^63^ |  |
| 17 | c.538-1G>C | Dubowitz-like syndrome | | | | | | | - | ^10^ |  |
