# Supplementary figures and images for: Autosomal recessive variants c.953A>C and c.97-1G>C in NSUN2 causing intellectual disability: a molecular dynamics simulation study of loss-of-function mechanisms
Source: Front Neurol. 2023 May 25;14:1168307. doi: 10.3389/fneur.2023.1168307 (PMC10249782; doi:10.3389/fneur.2023.1168307)

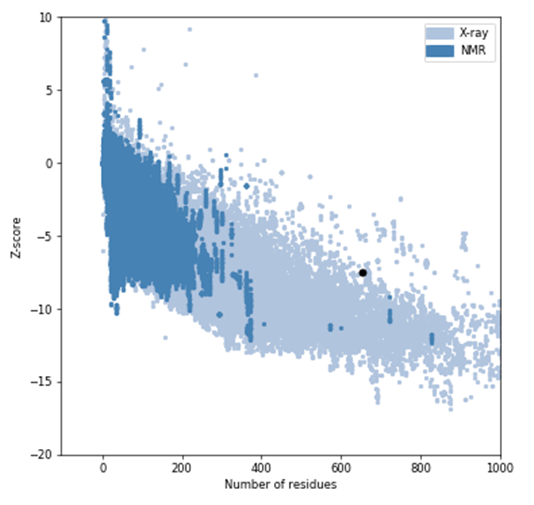

Supplement: Supplementary file 3 [file Image_1.PNG]

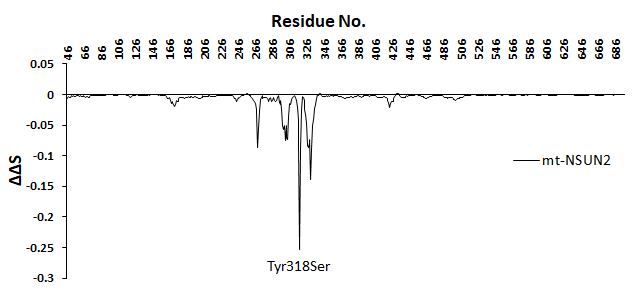

Supplement: Supplementary file 4 [file Image_2.PNG]
